# Nanodiamond-mediated delivery of microRNA-7 for the neuroprotection of dopaminergic neurons

**DOI:** 10.3389/fbioe.2024.1480573

**Published:** 2025-01-08

**Authors:** Yuping Han, Yue Yao, Xinyi Wen, Hao Wang, Shurong Li, Bingyin Su

**Affiliations:** Development and Regeneration Key Lab of Sichuan Province, Department of Histology and Embryology, Department of Pathology, Chengdu Medical College, Chengdu, China

**Keywords:** nanodiamonds, microRNA-7, Parkinson’s disease, dopaminergic neurons, oxidative stress

## Abstract

Parkinson’s disease (PD) is a neurodegenerative disorder characterized by the gradual loss of dopaminergic neurons in the substantia nigra and the accumulation of α-synuclein aggregates known as Lewy bodies. MicroRNA-7 (miR-7) targets the gene *SNCA*, which encodes α-synuclein, reducing its expression and alleviating neuronal damage in PD. Regulating the post-transcriptional levels of α-synuclein through miR-7 effectively inhibits its production. Herein, we use nanodiamonds as carriers to deliver miR-7 (N-7), which can effectively protect dopaminergic neurons. Dopaminergic neurons efficiently take up N-7 and express miR-7. N-7 inhibits the expression of α-synuclein, reduces oxidative stress and restores dopamine levels effectively. These findings suggest that nanocomposites have significant potential in treating PD.

## Graphical Abstract

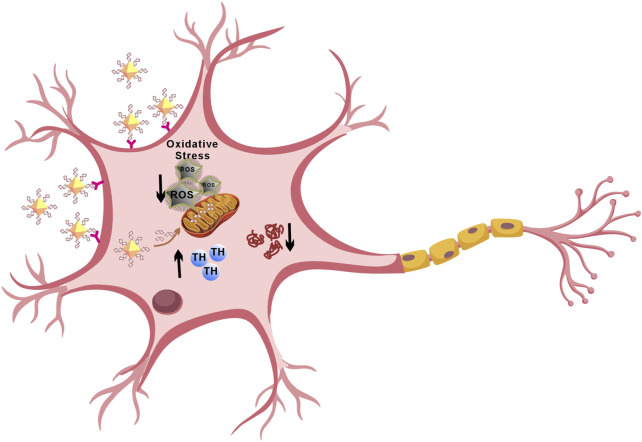

## 1 Introduction

Parkinson’s disease (PD) is a common neurodegenerative disorder that primarily affects the elderly. Its main pathological feature is the degeneration and death of dopaminergic neurons in the substantia nigra, as well as the formation of Lewy bodies, leading to clinical symptoms such as resting tremor, bradykinesia, and rigidity (Bloem et al., 2021). The primary component of Lewy bodies and Lewy neurites is aggregated α-synuclein (α-syn) (Aarsland et al., 2021). The pathogenesis of PD involves mutations and aggregation of α-synuclein, oxidative stress, and mitochondrial damage (Hansson, 2021). Mutations in the α-synuclein gene can lead to structural changes in the α-synuclein protein, causing dopaminergic neuron death (Iranzo et al., 2021). Environmental toxins like 1-methyl-4-phenyl-1,2,3,6-tetrahydro-pyridine (MPTP) and rotenone, along with mutations in genes like DJ-1 and PINK-1, cause mitochondrial damage, further contributing to dopaminergic neuron death (Malpartida et al., 2021). Oxidative stress and abnormal phosphorylation of proteins can also lead to misfolding and subsequent dopaminergic neuron death (Jomova et al., 2023). Clinically, treatments for PD include dopamine precursors such as levodopa, dopamine receptor agonists, monoamine oxidase inhibitors, anticholinergics, and excitatory amino acid inhibitors (Vijiaratnam et al., 2021). Levodopa remains the most effective drug for PD treatment but can lead to long-term side effects like dyskinesia (Ogawa et al., 2022). Current treatments only alleviate symptoms and do not address the underlying degenerative process.

Gene therapy targeting specific proteins could provide neuroprotection and restoration by correcting the underlying disease mechanisms. miR-7, an endogenous single-stranded non-coding small RNA of 23 nucleotides, targets the SNCA gene encoding α-synuclein (Barrie et al., 2018). Physiologically, miR-7 expression levels are inversely correlated with α-synuclein levels, maintaining them within physiological ranges (Zhang et al., 2021). Reduced levels of miR-7 in PD patients’ brains correlate with α-synuclein accumulation, DA neuron damage, and reduced striatal dopamine (McMillan et al., 2017). Our data suggest that miR-7 can reduce oxidative stress and inhibit SNCA gene translation, thereby protecting dopaminergic neurons. This suggests that targeted delivery of miR-7 to dopaminergic neurons may have therapeutic potential for PD.

However, naked miRNAs face several challenges due to their negative charge, off-target effects, short half-life in circulation, and instability. Cationic liposomes like Lipofectamine RNAiMAX have been used for gene therapy delivery but can be deactivated in the presence of serum (Tenchov et al., 2012) and are unstable during storage. Adeno-associated virus (AAV) can be used to deliver microRNA, but most often involves intracranial stereotactic injection. Nanoparticles are widely used in the biomedical industry due to their excellent physical and chemical properties (Gong et al., 2022; Cui et al., 2022; Meng et al., 2024; Xie and Xie, 2024). Numerous studies have reported the therapeutic effects of nanocarrier-RNA drug delivery systems for treating diseases, such as using polyethyleneimine, chitosan, silica nanoparticles, Au nanoparticles and cationic lipid nanoparticles to deliver RNA to target regions (Liu et al., 2023). From a delivery perspective, they have achieved expected outcomes in experimental settings; however, further investigation into drug targeting accuracy and long-term delivery safety is required. Nanodiamonds (NDs) possess ideal characteristics for nanocarrier functionality, including: nanoscale size, stable and inert core, tunable surface modifications, intrinsic fluorescence without photobleaching, minimal toxicity (Li et al., 2019) and ability to form complexes with drugs (Liu et al., 2015). Compared to other carbon-based nanomaterials, NDs have unique abilities to dissolve hydrophobic drugs, increasing stability and prolonging circulation time (Fujiwara et al., 2020). Given their inherent biocompatibility, structural stability, and nontoxic nature, NDs have broad applications in biomedicine and have been used to deliver various therapeutic molecules-including drugs, RNAs, hormones, proteins, and vitamins (Chen et al., 2018; Yang et al., 2020). Numerous studies have demonstrated their efficacy as drug carriers, such as for paclitaxel and cetuximab in the treatment of colorectal cancer (Lin et al., 2017), G9a inhibitors for hepatocellular carcinoma (Gu et al., 2019), doxorubicin for glioblastoma (Li et al., 2017), prostate cancer (Salaam et al., 2014), and alendronate for osteoporosis (Ahn et al., 2021), showing superior therapeutic effects compared to the use of the drugs alone.

To overcome the challenges of delivering miR-7 to dopaminergic neurons, we propose using NDs as carriers to create a nanocomplex (N-7) for PD treatment. Results show that N-7 has good biocompatibility. Dopaminergic neurons rapidly take up N-7 and express miR-7. Furthermore, the transfection efficiency of miR-7 when complexed with NDs outperforms that of Lipofectamine RNAiMAX. Importantly, N-7 can rescue the loss of tyrosine hydroxylase (TH) in PD cell models. Further research indicates that the therapeutic effect of N-7 is achieved through inhibition of abnormal α-synuclein aggregation, promotion of TH expression, and antioxidant activity. In summary, these findings demonstrate the potential of N-7 for neuroprotective effects in PD treatment.

## 2 Materials and methods

### 2.1 Materials

Nanodiamonds were purchased from Sigma-Aldrich (USA). miR-7 was obtained from GenePharma (Shanghai, China). SH-SY5Y, MN9D and HEK293T cell lines were purchased from Cell Bank of Chinese Academy of Sciences (Shanghai, China). Dulbecco’s Modified Eagle’s Medium (DMEM), Dulbecco’s Modified Eagle Medium/Nutrient Mixture F-12 (DMEM/F12) and fetal bovine serum (FBS) were from Gibco, ThermoFisher. Cell Counting Kit-8 was purchased from Beyotime (Shanghai, China). The miR-7 used in the article is an artificially synthesized short double-stranded RNA. The sequence of miR-7 and NC are in Supplementary Table S1.

### 2.2 Cell culture

SH-SY5Y and MN9D cells were cultured in DMEM/F12 medium, and HEK293T cells were cultured in DMEM medium. Both media were supplemented with 10% heat-inactivated fetal bovine serum (FBS) and antibiotics (1% streptomycin and 1% penicillin). Cells were maintained at 37°C in a humidified atmosphere with 5% CO_2_.

### 2.3 Preparation of N-7

#### 2.3.1 NDs and miR-7 complex ratio

We used RNase-free water to dilute NDs (10 mg/mL) 10–100 times and mix them with miR-7 at mass ratios of NDs:miR-7 of 0, 0.5, 1, 2, 5, 5, 10, 20, 30. Leave the mixture at room temperature for 30 min after mixing uniformly. Set the naked miR-7 as the control group. After 30 min, load the samples onto an agarose gel for electrophoresis.

#### 2.3.2 NDs and miR-7 complexation time

Mix NDs and miR-7 at the optimal ratio determined in the previous step. Set different incubation times: 2 h, 1 h, 30 min, 15 min, 5 min, 1 min, and 0 min. Set the naked miR-7 and naked NDs as the control groups. Load all samples simultaneously onto an agarose gel for electrophoresis.

#### 2.3.3 Complex strength of NDs and miR-7

Prepare two sets of NDs-miR-7 complexes at the optimal ratio and incubation time determined in steps 1 and 2. Centrifuge one set of complexes using a simple mini centrifuge for 1 min and then load the supernatant. Load the other set without centrifugation. Set the supernatants of naked miRNA and empty NDs after centrifugation as the control groups. Load all samples onto an agarose gel for electrophoresis.

#### 2.3.4 Drug loading efficiency

Drug Loading Efficiency (%) = m (miR-7) / [m (miR-7) + m (NDs)] × 100%

### 2.4 Agarose gel electrophoresis

Prepare a 1% agarose gel. Add 1× TAE electrophoresis buffer to completely immerse the gel. In the top left well, add 5 μL of marker. Mix RNA samples with loading buffer and sequentially add them to the wells. Place the gel with the sample side towards the negative electrode and run at 100 V for approximately 20 min. Place the gel in a gel imaging system, set parameters, and perform imaging. miRNA should appear as a single band.

### 2.5 Dynamic light scattering (DLS)

Dilute the samples in DEPC-treated water to an appropriate concentration and measure at 25°C. Fit the correlation function using the cumulant method to obtain the hydrodynamic diameter (Z-average, nm) and its polydispersity index (PDI). Repeat three times.

### 2.6 Transmission electron microscopy (TEM)

Place a copper grid coated with a continuous carbon film on a 10 μL drop of sample solution and leave it for 5 min to adsorb the nanomaterial. Stain the grid by placing it in an equal volume of 2% phosphotungstic acid for 2 min, then dry it. Observe the morphology under TEM. Use ImageJ software for size analysis.

### 2.7 Biocompatibility analysis

We set up a blank group (NDs), a control group (miR-7), and experimental groups with varying mass ratios of NDs to miR-7 (0–30) and different mixing times (0–120 min). After mixing NDs and miR-7 for 10 min, the mixture was centrifuged at 6,000 rpm for 1 min, and the supernatant was collected. SH-SY5Y cells in the logarithmic growth phase were digested with 0.25% trypsin and seeded at 5 × 10^4 cells per well in a 96-well plate, with five replicates per group, and incubated overnight. The culture medium was then removed. The blank group received no treatment, the control group had its medium replaced with fresh culture medium, and the experimental groups were treated with different concentrations of NDs (0, 5, 25, 50, 100, 200 μg/mL) and miR-7 (0, 5, 10, 50, 100 mM), as well as N-7 complexes and other groups (Ctrl, miR-7, NC, L-7, L-NC, N-NC) (with miR-7 at 10 nM, where NC is a negative control miRNA). Solutions were prepared by mixing NDs and miR-7 for N-7, and Lipofectamine RNAiMAX and miR-7 for L-7, followed by incubation and addition to the cell culture medium. Cells were further incubated for 48 h. A CCK-8 assay was performed by adding 100 μL of a 10% CCK-8 reagent mixture to each well, incubating in the dark for 1–2 h until the medium turned orange-red, and measuring absorbance at 450 nm. Cell viability was calculated using the formula: Cell Viability (%) = (A (Experimental Group) − A (Blank Group)) / (A (Control Group) − A (Blank Group)) × 100%.

### 2.8 Confocal fluorescence microscopy

Cells expressing fluorescent protein markers or cells immunostained for microtubule were imaged using confocal microscope. CY5 was excited with a 561 nm helium-neon laser and Hoechst 33258-labeled nuclei were excited with a 405 nm diode laser, respectively. The imaging channels were set at 570–620 and 450–500 nm, respectively.

### 2.9 Western blot

Separate protein samples using SDS-PAGE (Sodium Dodecyl Sulfate Polyacrylamide Gel Electrophoresis). Transfer the proteins onto a PVDF (Polyvinylidene Fluoride) membrane. Block the membrane with 5% non-fat milk in Tris Buffered Saline with Tween 20 (TBST) at room temperature. Incubate with primary antibodies overnight at 4°C. Wash the membrane with PBS (Phosphate Buffered Saline). Incubate with the secondary antibody conjugated to a reporter molecule at room temperature for 2 h. Visualize the proteins using an enhanced chemiluminescence kit.

### 2.10 Flow cytometry

Before measurements, cell media was removed and cells were washed for three times with PBS. Next, 0.2 mL trypsin (Invitrogen) was added to each sample and incubated for 1 min at 37°C before 0.5 mL DMEM/F12 was added. Cell suspensions were transferred into tubes before analyzed using a FACS Calibur flow cytometer. Consistent gating based on cell size and granularity (forward and side scatter) was applied to select the fluorescence signals of counted cells. At least 10,000 cells were counted for each sample and experiments were performed in triplicates.

### 2.11 qRT-PCR analysis

Extract RNA from cells using Trizol reagent. Following the instructions provided with the Vazyme miRNA 1st Strand cDNA Synthesis Kit (by stem-loop), reverse transcribe RNA into cDNA. Conduct real-time quantitative PCR using the Vazyme miRNA Universal SYBR qPCR Master Mix kit according to the manufacturer’s instructions. The primer sequences used are detailed in Supplementary Table S2.

### 2.12 Detection of reactive oxygen species (ROS) levels

Dilute DCFH-DA in serum-free medium to a final concentration of 10 μM. Remove the cell culture medium from adherent cells in the dish and add 200 μL of the diluted probe to cover the cells fully. For the positive control group, add 0.2 μL of Rosup along with the probe. Incubate at 37°C for 20 min. Stain with Hoechst 33342 (1:100 dilution) for 10 min. Gently wash the cells three times with serum-free medium, leaving a small amount of medium on the culture insert to keep it moist. Observe under a confocal laser scanning microscope with excitation wavelengths of 350 nm (for Hoechst 33342) and 488 nm (for DCF).

### 2.13 Statistical analysis

Data analysis and statistical graph creation were performed using GraphPad Prism 8 software. Independent samples t-tests were used to compare differences between two groups. One-way ANOVA was used to compare differences among groups. Data are presented as mean ± standard deviation. A *p*-value less than 0.05 indicates statistically significant difference (*P < 0.05; **P < 0.01; ***P < 0.001).

## 3 Results and discussion

### 3.1 Preparation, characterization and biocompatibility of N-7

**FIGURE 1:**
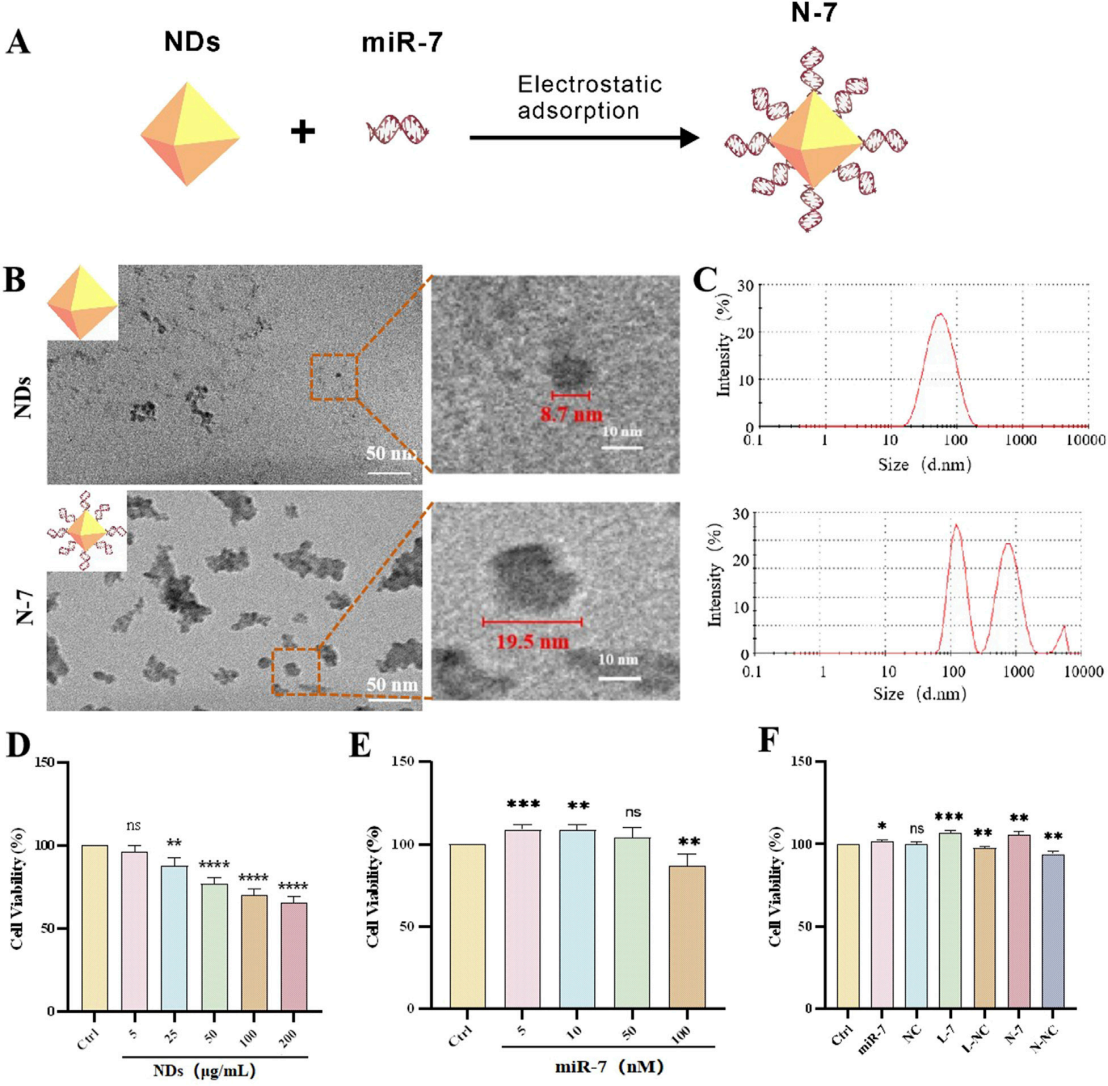
Characterization and biocompatibility of NDs and N-7. **(A)** Schematic diagram illustrates the synthesis of the N-7 complex using the electrostatic adsorption method. **(B)** Transmission Electron Microscopy of NDs and N-7. **(C)** Dynamic light scattering analysis of NDs and N-7. CCK-8 detection of the effects of different concentrations of NDs **(D)** or miR-7 **(E)** on the cell viability of SH-SY5Y cells (n = 3). **(F)** CCK-8 detection of the effects on the cell viability of SH-SY5Y cells under different conditions. NDs, nanodiamonds. N-7, the complex of nanodiamonds and microRNA-7. NC, nonsense microRNA. L-7, Lipofectamine RNAiMAX transfection of microRNA-7 (n = 3). L-NC, Lipofectamine RNAiMAX transfection of nonsense microRNA. N-NC, the complex of nanodiamonds and nonsense microRNA. ns means no statistical significance, *P < 0.05, **P < 0.01, ***P < 0.001 and ****P < 0.0001 with respect to the control group.

We conjugated miR-7 to the NDs through electrostatic adsorption (Figure 1A). Gel retardation experiments showed that when the mass ratio of NDs to miR-7 is 20:1, miR-7 is completely bound to the NDs (Supplementary Figure S1A), with a drug loading of 4.7 wt.%. Binding is complete within 10 min of co-incubation, whereas when NDs mixed with miR-7 are immediately loaded onto the gel, very little miR-7 binds to the NDs (Supplementary Figure S1B). After centrifugation of the synthesized N-7 complex, agarose gel electrophoresis of the supernatant did not show any miR-7 (Supplementary Figure S1C), indicating that miR-7 is stably bound to the NDs.

Transmission electron microscopy (TEM) images show that NDs appear as spherical particles with some aggregation, with diameters of approximately 5–10 nm. After complexing with miR-7, the solution becomes more viscous, and the material appears as aggregated clusters with scattered particles around them, with diameters of approximately 15–20 nm (Figure 1B). Dynamic light scattering (DLS) measurements show an average particle size of 47.6 nm for NDs with a polydispersity index (PDI) of 0.23; after complexing with miR-7, the particle size increases to 225.4 nm with a PDI of 0.77 (Figure 1C). The difference in particle sizes measured by DLS and TEM may be due to the hydrated state of the particles in DLS measurements and possible aggregation of NDs in the liquid medium. DLS results also show that the zeta potential of NDs decreases from 31.3 mV to 22.2 mV after complexing with miR-7 (Supplementary Figure S2). The positive zeta potential of the N-7 complex facilitates interaction with negatively charged components of the cell membrane, such as heparan sulfate proteoglycans, promoting uptake through endocytosis, phagocytosis, or pinocytosis.

Compared to the control group, the cell survival rate gradually decreases with increasing concentrations of NDs, but the survival rate remains above 50% across all tested concentrations; low concentration NDs (5 μg/mL) have almost no effect on cell survival (Figure 1D). These results indicate that nanodiamonds possess good biocompatibility, as shown in other studies (Chen et al., 2018). Liposome-transfected miR-7 at low concentrations (5 nM, 10 nM, 50 nM) does not harm cell viability and is even beneficial at 5 nM and 10 nM (Figure 1E). N-7 nanocomplexes and L-7 (miR-7 concentration 10 nM) significantly increase cell survival rates after 48 h of co-incubation with SH-SY5Y cells. In comparison, free miR-7 benefits cell survival, while NC has no effect, and both liposome transfection and ND-mediated delivery slightly decrease cell survival (Figure 1F). These data suggest that N-7 complexes protect dopaminergic neurons.

### 3.2 The internalization of NDs both *in vitro* and *in vivo*

**FIGURE 2:**
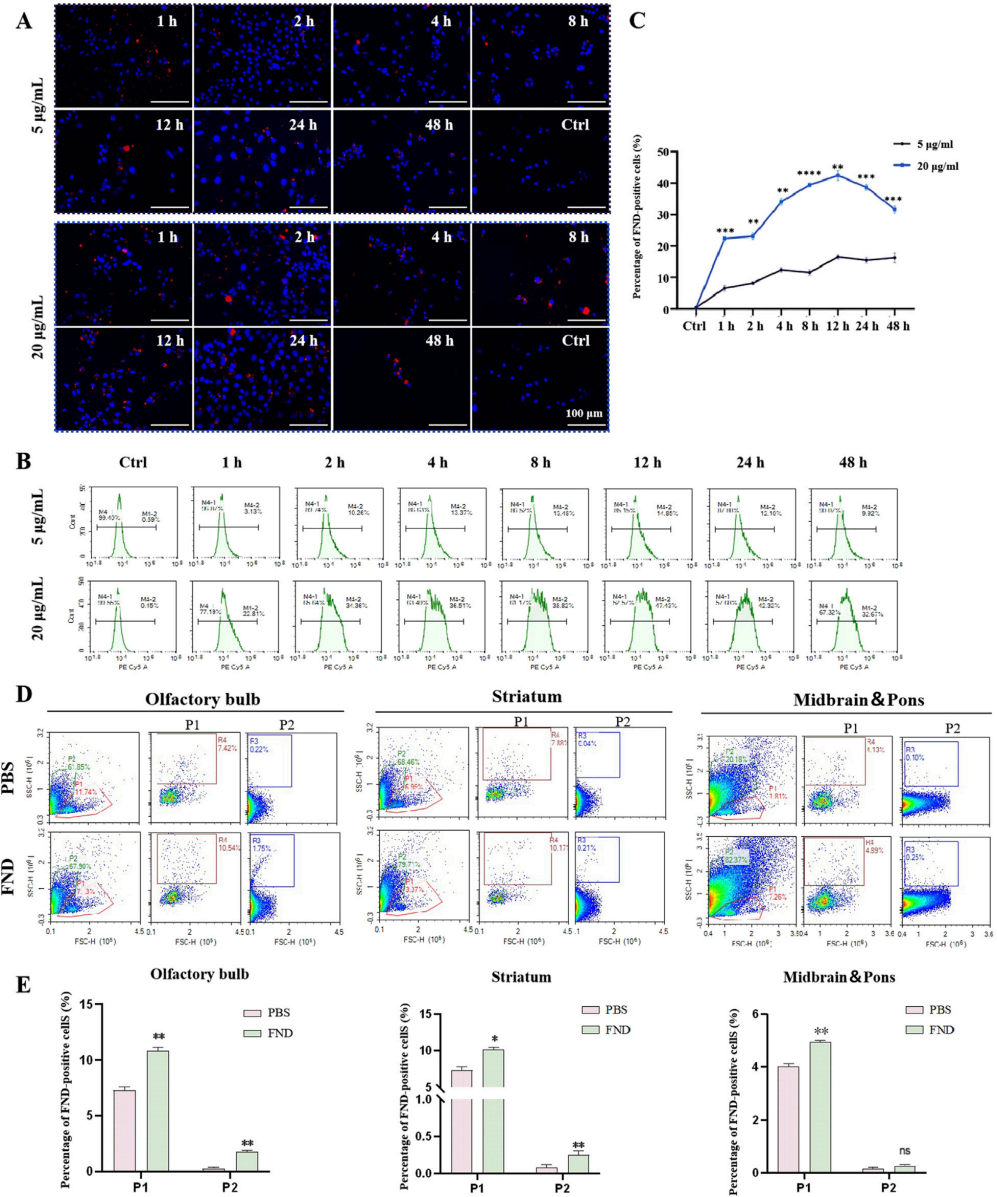
The internalization of NDs both *in vitro* and *in vivo*. **(A)** MN9D cells were incubated with different concentrations of NDs for up to 48 h. At indicated time points, intracellular fluorescence was observed using confocal laser scanning microscopy. **(B)** and **(C)** Fluorescence of internalized NDs were quantified using flow cytometry analysis (n = 3). **P < 0.01, ***P < 0.001 and ****P < 0.0001 with respect to the control group. **(D)** and **(E)** Flow cytometric analysis of the distribution of fluorescent nanodiamonds in the olfactory bulb, striatum, midbrain and pons (n = 3). P1 represents neurons, and P2 represents glial cells. ns means no statistical significance, *P < 0.05 and **P < 0.01 with respect to the PBS group.

Confocal laser scanning microscopy was used to observe the uptake of red fluorescent NDs by MN9D cells at different time points ranging from 1 h to 48 h, and at both low (5 μg/mL) and high (20 μg/mL) concentrations. (Figure 2A). At a low concentration (5 μg/mL), NDs are observed to be evenly distributed in the cells after 1 h of incubation, with a tendency to accumulate around the nucleus at 8 h, and remain in the perinuclear region up to 48 h. At a high concentration (20 μg/mL), NDs are seen to accumulate around the nucleus after 8 h, and remain in the cytoplasm up to 48 h, although fewer cells are visible at this time point.

Flow cytometry results (Figures 2B, C) show that the uptake rate of NDs by MN9D cells at a low concentration (5 μg/mL) increases gradually from 1 to 4 h, stabilizes from 4 to 24 h, and then decreases, with 9.34% of cells having internalized NDs at 48 h. At a high concentration (20 μg/mL), the uptake rate is faster, reaching 21.98% at 1 h, with the highest uptake (46.59%) at 12 h, followed by a gradual decline to 31.86% at 48 h. This indicates that NDs are taken up by cells in a concentration-dependent and time-dependent manner, and that over time, nanodiamonds may be expelled from cells via exocytosis. Nanodiamonds were administered intranasally to mice, and their distribution in the olfactory bulbs and striatum was detected using flow cytometry (Figures 2D, E). Thus, nanodiamonds can be internalized both *in vitro* and *in vivo*.

### 3.3 NDs-mediated cellular uptake and expression of miR-7

**FIGURE 3:**
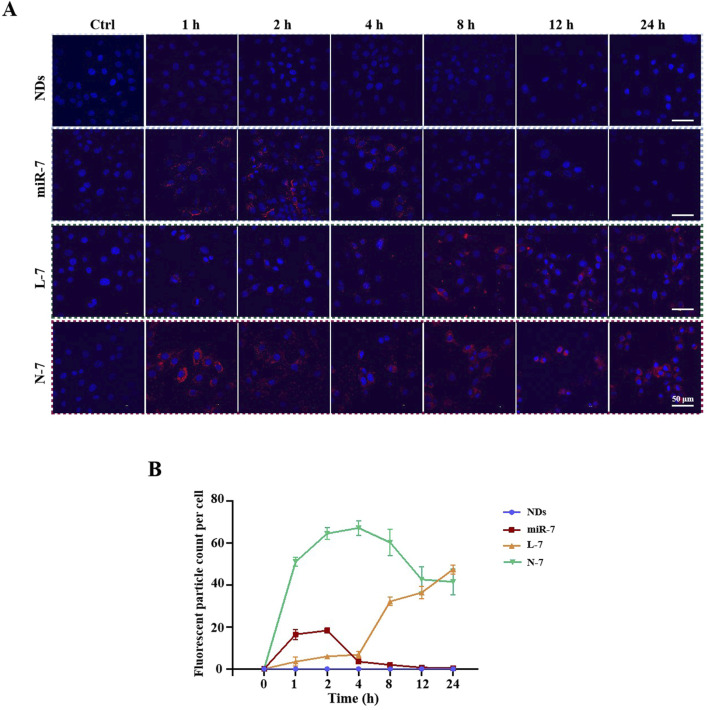
Uptake of N-7 by MN9D cells. **(A)** Imaging the uptake of Cy5-miR-7 by MN9D cells under different conditions using confocal laser scanning microscopy. **(B)** Fluorescent particle count per cell (n = 20).

Cy5-labeled miR-7 (Cy5-miR-7) was transfected into MN9D cells using NDs and Lipofectamine RNAiMAX. Confocal laser scanning microscopy observations (Figure 3) showed that naked Cy5-miR-7 displays strong red fluorescence in cells from 1 to 4 h post-incubation, with a tendency to accumulate around the nucleus at 2 h and decreasing fluorescence intensity at 4 h. For Lipofectamine RNAiMAX-transfected Cy5-miR-7 (L-7), the intensity of red fluorescence increases over time, peaking at 24 h. For Cy5-miR-7 complexed with NDs (N-7), strong red fluorescence is observed from 1 to 24 h, with a significant amount of punctate accumulation in the cytoplasm at 1 h, and stable fluorescence intensity from 2 to 24 h.

**FIGURE 4:**
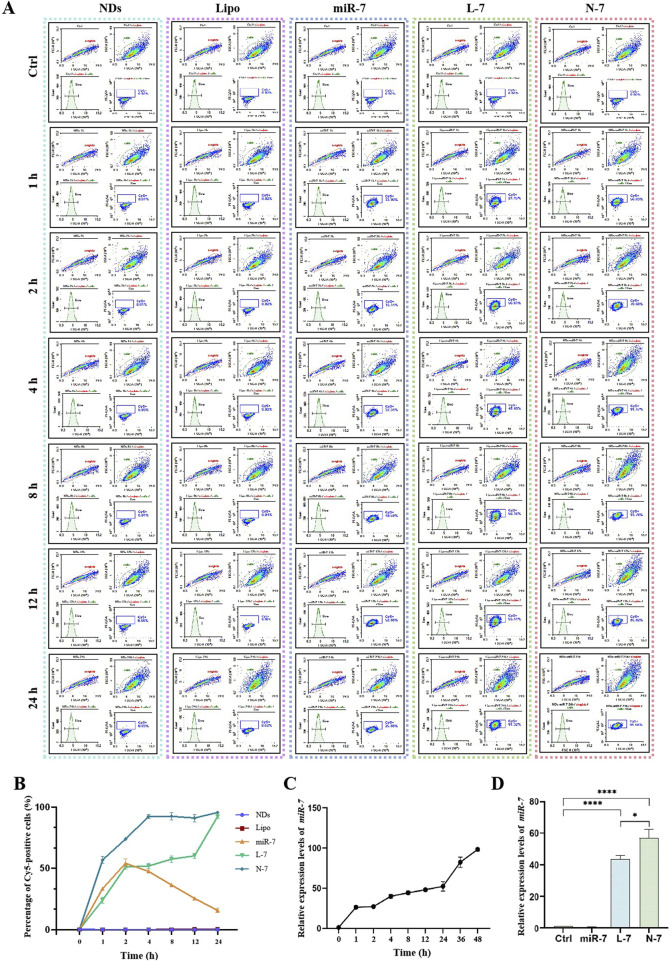
Expression of N-7 by MN9D and SH-SY5Y cells. **(A)** and **(B)** Fluorescence of internalized NDs by MN9D cells were quantified using flow cytometry analysis. **(C)** Relative expression levels of miR-7 in SH-SY5Y cells at different times post-transfection with Lipofectamine RNAiMAX. **(D)** Relative expression levels of miR-7 in SH-SY5Y cells after 24 h of incubation with naked miR-7, L-7, and N-7. U6 as the internal reference gene in **(C)** and **(D)** (n = 3). *P < 0.05, ****P < 0.0001.

Flow cytometry analysis (Figures 4A, B) showed that neither NDs nor Lipofectamine RNAiMAX alone display any fluorescence signal. Naked Cy5-miR-7 shows an increase in the number of Cy5-positive cells from 1 to 8 h, peaking at 8–12 h and declining at 24 h, with only about 50% of cells being Cy5-positive at the peak. For Lipofectamine RNAiMAX-transfected Cy5-miR-7, the number of Cy5-positive cells increases to about 50% at 1–2 h and remains stable until 12 h, reaching a peak of approximately 90% at 24 h. For NDs-mediated Cy5-miR-7, over 50% of MN9D cells are Cy5-positive at 1 h, and the uptake continues to rise until it reaches a maximum of 96% at 24 h.

The expression of miR-7 within cells increases over time after transfection with Lipofectamine RNAiMAX (Figure 4C). Naked miR-7 co-incubated with SH-SY5Y cells for 24 h does not differ significantly in relative expression compared to the control group, while L-7 and N-7 co-incubated with SH-SY5Y cells for 24 h show significantly higher miR-7 expression. Furthermore, miR-7 complexed with NDs demonstrates superior transfection efficiency compared to Lipofectamine RNAiMAX, with statistically significant differences (*P* < 0.05) (Figure 4D).

Studies have shown that NDs are primarily internalized via clathrin-mediated endocytosis and can bind serum proteins to enhance receptor-mediated endocytosis (Igarashi et al., 2012). Using polyethylenimine-modified diamond nanoparticles (50 nm) increased DNA delivery efficiency by 70-fold. This suggests that nucleic acids carried by NDs can be efficiently taken up by cells. In this study, we confirmed that Cy5-labeled miRNAs complexed with NDs enter cells rapidly, with over 50% of cells showing Cy5 fluorescence after 1 h, reaching 90% by 4 h and maintaining this level up to 24 h. Compared to Lipofectamine RNAiMAX, NDs deliver miR-7 faster and achieve 1.5 times higher intracellular expression of miR-7 after 24 h.

### 3.4 N-7 targets the mRNA of α-synuclein

**FIGURE 5:**
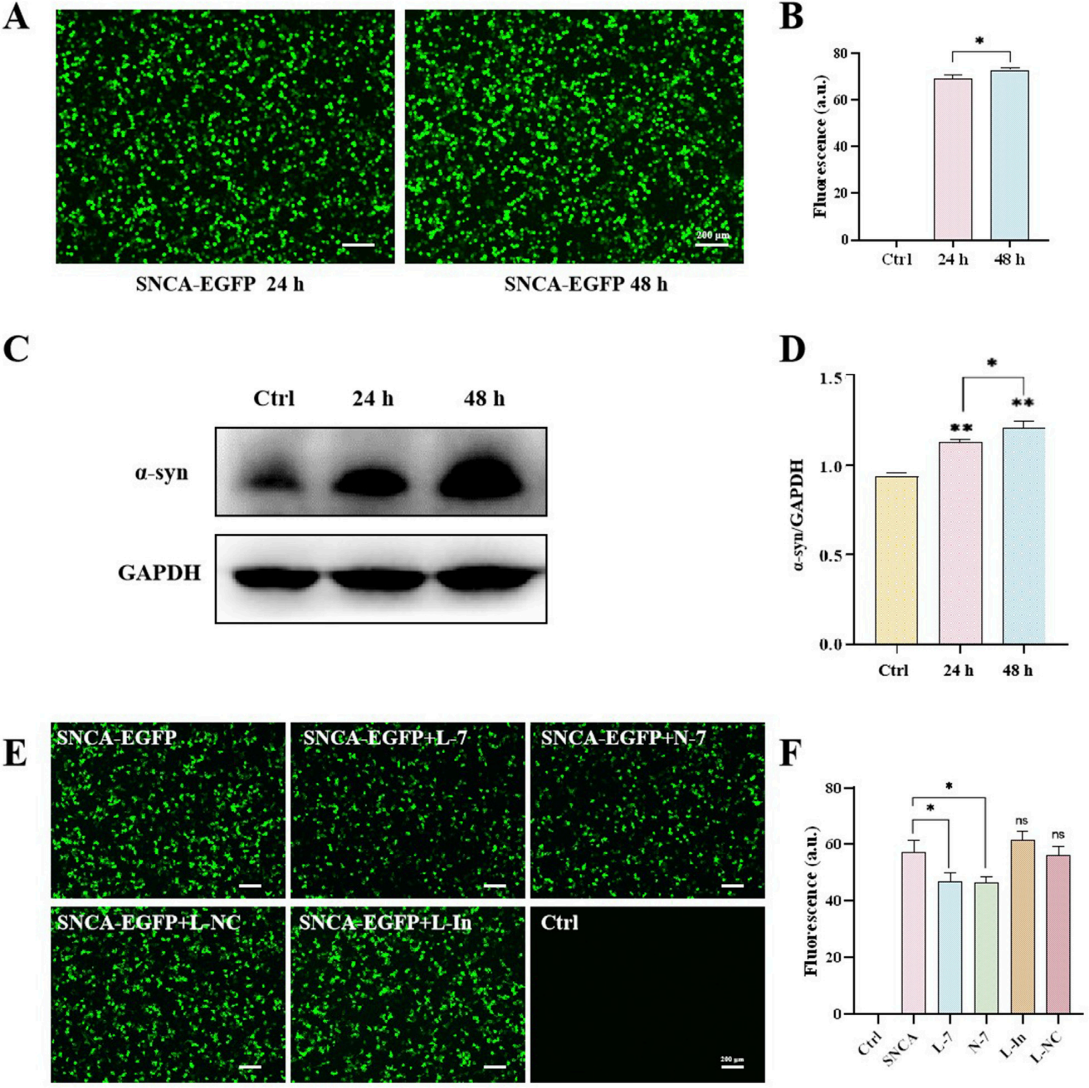
N-7 targets the mRNA of α-synuclein. **(A)** Protein levels of α-synuclein were analyzed by Western blotting and its intensity is quantified in **(B)**. **(C)** EGFP fluorescence in HEK293T cells after transfection with the SNCA-EGFP plasmid for 24 or 48 h and quantified in **(D)**. **(E)** EGFP fluorescence in HEK293T cells under different conditions and quantified in **(F)**. L-In, Lipofectamine RNAiMAX transfection of mimic miR-7 inhibitors. ns means no significant difference, *P < 0.05 and **P < 0.01.

The motor symptoms of PD are largely due to the loss of dopaminergic neurons in the substantia nigra and the resulting dysregulation of basal ganglia activity (Russo et al., 2021). Neuropathologically, PD is characterized by protein inclusions known as Lewy bodies (LBs), with α-synuclein being the major protein component of these inclusions (Cascella et al., 2021). To examine the effect of N-7 on *SNCA* expression, we transfected the human embryonic kidney cell line 293T (HEK293T) with the pLenti-DsRed_IRES_SNCA:EGFP plasmid using TransIntro EL reagent. After 24 h of transfection, a significant number of cells expressing EGFP green fluorescence were observed under the microscope. The expression level of α-synuclein was found to be approximately 65 times higher than in the control group. By 48 h post-transfection, both the fluorescence intensity and α-synuclein expression had further increased, reaching about 70 times that of the control (Figures 5A, B). This indicates the successful establishment of an α-synuclein overexpression model. Western blot analysis revealed an increase in α-synuclein protein expression levels 24 h post-transfection with the SNCA-EGFP plasmid. Additionally, a significant enhancement in fluorescence intensity was observed at the 48-h mark following transfection (Figures 5C, D).

After overexpressing or inhibiting miR-7 for 24 h and then transfecting the SNCA-EGFP plasmid for another 24 h, the expression of EGFP green fluorescence was observed under confocal microscopy in HEK293T. Approximately 50% of the cells expressed SNCA-EGFP in the SNCA-EGFP group. Both the Lipofectamine RNAiMAX-transfected miR-7 overexpression (L-7) group and the nanodiamond-loaded miR-7 (N-7) group showed a decrease in SNCA-EGFP fluorescence expression. Cells treated with the negative control Lipofectamine RNAiMAX-transfected non-targeting miRNA (L-NC) showed no significant changes in fluorescence expression. Cells treated with the Lipofectamine RNAiMAX-transfected miR-7 mimic inhibitor (L-In) showed a slight increase in fluorescence expression, but the difference was not statistically significant (Figures 5E, F), demonstrating the suppressive effect of miR-7 on *SNCA* gene translation.

### 3.5 Protection of dopaminergic neurons by N-7

1-Methyl-4-phenylpyridinium (MPP^+^) is an inhibitor of mitochondrial complex I that leads to the depletion of cellular ATP and loss of membrane potential, ultimately causing mitochondrial dysfunction and increased production of ROS (Prasad and Hung, 2020). It is commonly used as an inducer for PD cell models. By exposing SH-SY5Y cells to a gradient of MPP^+^ concentrations and analyzing their cell viability, the optimal experimental concentration for creating PD models was determined. Results showed that cell viability decreased with increasing MPP^+^ concentration; at 100 μM MPP^+^, cells exhibited damage and death, which was statistically significant; at 1 mM MPP^+^, there was a substantial cell death, with a survival rate of approximately 50%, reaching the half-maximal lethal concentration, suggesting that this concentration could be used as the MPP^+^ treatment dose in this study (Supplementary Figure S3).

**FIGURE 6:**
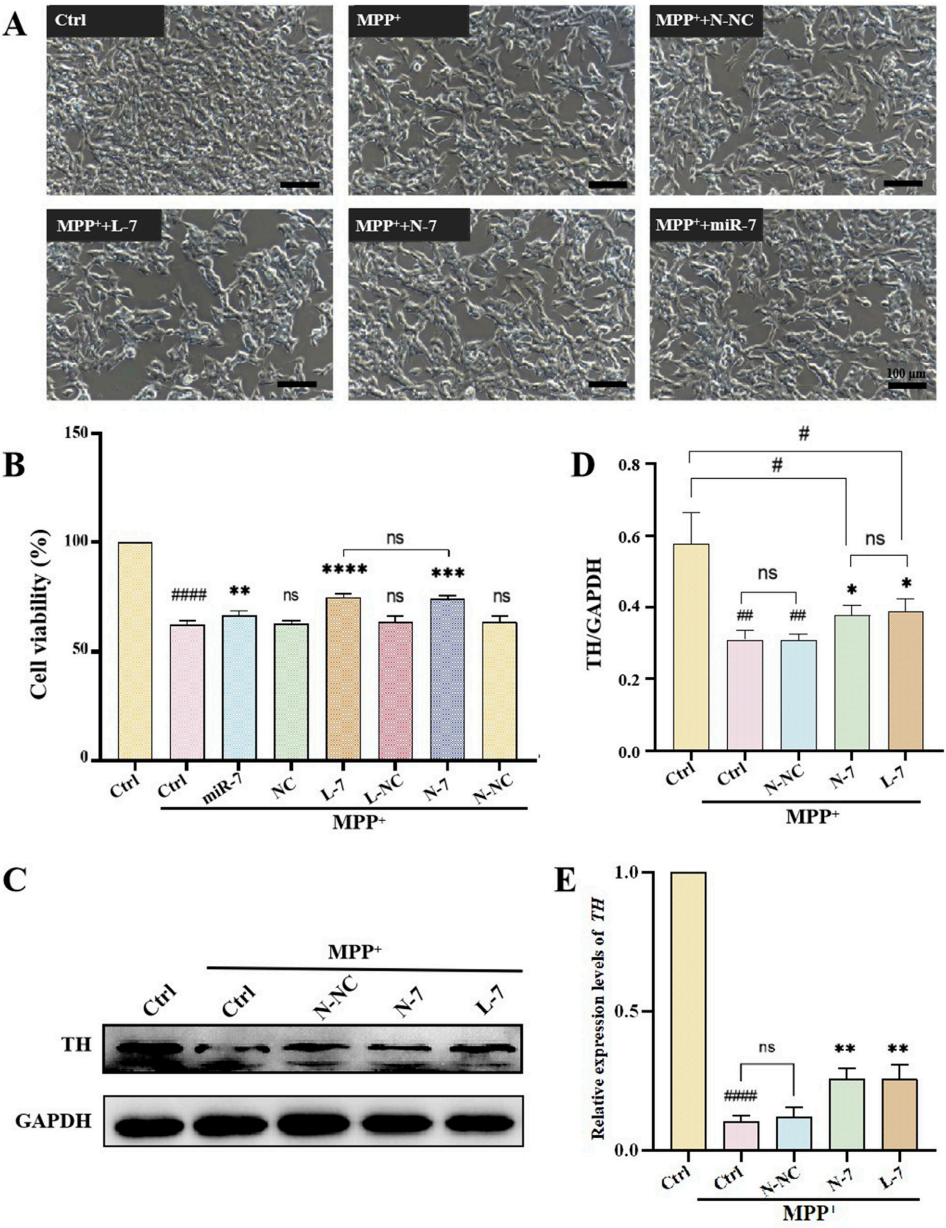
The effect of N-7 on MPP^+^-induced PD model. **(A)** SH-SY5Y cells were photographed with an inverted microscope in white light. **(B)** CCK-8 detection of the effects on the cell viability of SH-SY5Y cells under different conditions (n = 3). ^####^ *P* < 0.0001 with respect to the control group. ns means no statistical significance, **P < 0.01, ***P < 0.001 and ****P < 0.0001 with respect to the MPP^+^ Ctrl group. **(C)** Protein levels of TH were analyzed by Western blotting and quantified in **(D)** (n = 3). ^##^ *P* < 0.01 with respect to the control group. *P < 0.05 with respect to the MPP^+^ Ctrl group. **(E)** qRT-PCR analysis of relative expression levels of TH (n = 3). ^####^ *P* < 0.0001 with respect to the control group. **P < 0.01 with respect to the MPP^+^ Ctrl group.

An important neurochemical abnormality in PD is the degeneration of dopaminergic neurons in the substantia nigra, leading to reduced striatal dopamine (DA) levels. TH is the rate-limiting enzyme of DA biosynthesis, catalyzing the hydroxylation of tyrosine to L-DOPA (Du et al., 2022). After 48 h of 1 mM MPP^+^ treatment, the cell viability of SH-SY5Y cells was approximately 60%. Treatment with naked miR-7 led to a significant recovery in cell viability (*P* < 0.01). Cells treated with NC, L-NC, or N-NC had cell viabilities similar to the model group, showing no therapeutic effect. After treatment with L-7, cell viability significantly increased compared to the model group (*P* < 0.0001), and after treatment with N-7, cell viability also significantly increased (*P* < 0.001); there was no statistical difference between the L-7 and N-7 groups (Figure 6B). The results indicate that N-7 does not induce changes in the density or morphology of SH-SY5Y cells.

After pre-treatment with N-NC, L-7, or N-7 for 12 h, MPP^+^ was added to achieve a final concentration of 1 mM, and samples were collected after 24 h for observation under inverted microscopy. Results showed that the control group had a high cell density with neurite-like cell projections; after MPP^+^ treatment, cell density decreased to approximately 50%, with no significant change in cell morphology; the density of treated cells did not change significantly, and cell morphology remained unchanged (Figure 6A). Western blot results showed that TH protein levels significantly decreased in the PD model group (*P* < 0.0001), while TH protein levels did not change in the negative control (N-NC) group. TH protein levels significantly increased in the positive control (L-7) group (*P* < 0.01), and the increase in the N-7 group (*P* < 0.01) was slightly higher than in the L-7 group (Figures 6C, D). qRT-PCR results were similar to those of the Western blot (Figure 6E). These data indicate that the N-7 nanocomplex not only prevents the MPP^+^-induced decrease in TH protein levels but also prevents the MPP^+^-induced decrease in TH RNA levels, demonstrating protective effects on dopaminergic neurons.

In this study, we used MPP^+^ to induce a PD cell model and used nanodiamonds (NDs) loaded with miR-7 to target the SNCA gene, providing protection against PD cell models. The results show that N-7 can improve the viability of cells damaged by MPP^+^ and can increase TH protein expression and RNA levels, demonstrating protective effects on dopaminergic neurons.

### 3.6 Reduction of cellular oxidative stress levels by N-7

**FIGURE 7:**
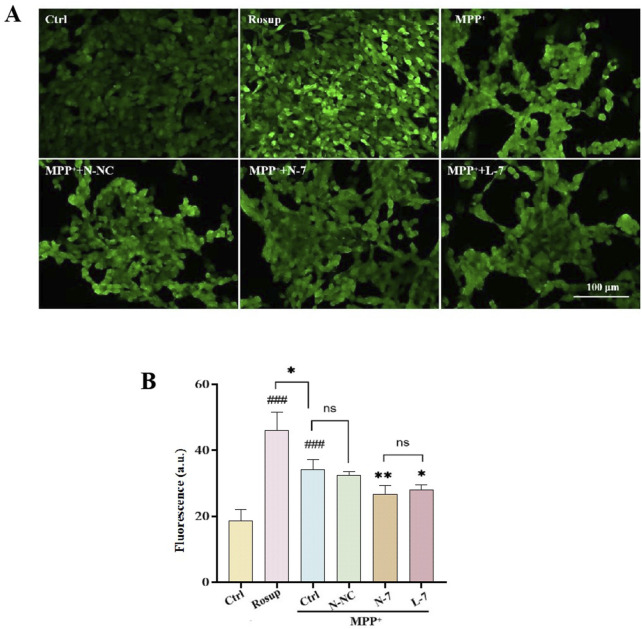
N-7 inhibits MPP^+^-induced oxidative stress in SH-SY5Y cells. **(A)** DCFH-DA probe fluorescence was imaged under different conditions using confocal laser scanning microscopy. **(B)** Statistical analysis of the average fluorescence intensity in Figure **(A)**, with Rosup serving as the positive control. ^###^ *P* < 0.001 with respect to the control group. *P < 0.05 and **P < 0.01 with respect to the MPP^+^ Ctrl group.

Oxidative stress is a prominent feature of PD and is closely associated with neuronal death and neurological dysfunction, playing a key pathogenic role in PD (Gao et al., 2024). Excessive generation of ROS caused by oxidative stress can lead to apoptosis of dopaminergic neurons, which is one of the primary causes of PD (Fan et al., 2023). As a complex I inhibitor, MPP^+^ inhibits ATP production and stimulates the formation of superoxide and peroxynitrite, thus damaging proteins through oxidative and nitrosative stress (Ghosh et al., 2016). Therapies aimed at reducing cellular ROS levels may provide neuroprotective treatment for PD. In the experiment, we used the DCFH-DA probe to detect intracellular ROS levels. Results showed weak ROS in the control group, high fluorescence intensity and high ROS levels in the positive control group (Rosup). After MPP^+^ treatment, ROS levels significantly increased compared to the control group. After treatment with N-NC, there was no significant change in ROS levels. After treatment with N-7 and L-7, ROS levels decreased, and there was no statistical difference between N-7 and L-7 (Figures 7A, B).

## 4 Conclusion

In this study, we designed a nanocomplex (N-7) for the treatment of PD. Compared to traditional RNA transfection reagents like Lipofectamine RNAiMAX, nanodiamonds (NDs) were able to more rapidly and efficiently deliver miR-7 to dopaminergic neurons. In MPP^+^-induced PD cell models, N-7 exhibited neuroprotective effects, not only improving the survival rate of dopaminergic neurons in PD models but also alleviating the pathological reduction in TH levels, inhibiting α-synuclein expression, and reducing oxidative stress. N-7 demonstrated excellent biocompatibility. In summary, the results of this study indicate that N-7 represents a promising strategy for drug delivery in the treatment of PD and other neurodegenerative diseases.

## Data Availability

The raw data supporting the conclusions of this article will be made available by the authors, without undue reservation.

## References

[B1] Aarsland D. Batzu L. Halliday G. M. Geurtsen G. J. Ballard C. Chaudhuri K. R. (2021). Parkinson disease-associated cognitive impairment. Nat. Rev. Dis. Prim. 7 (1), 47. 10.1038/s41572-021-00280-3 34210995

[B2] Ahn G. Y. Kim S. E. Yun T. H. Choi I. Park D. Choi S. W. (2021). Enhanced osteogenic differentiation of alendronate-conjugated nanodiamonds for potential osteoporosis treatment. Biomaterials Res. 25 (1), 28. 10.1186/s40824-021-00231-9 PMC8461989 34556181

[B3] Barrie E. S. Lee S. H. Frater J. T. Kataki M. Scharre D. W. Sadee W. (2018). Alpha-synuclein mRNA isoform formation and translation affected by polymorphism in the human *SNCA* 3′UTR. Mol. Genet. and Genomic Med. 6 (4), 565–574. 10.1002/mgg3.407 29730891 PMC6081226

[B4] Bloem B. R. Okun M. S. Klein C. (2021). Parkinson’s disease. Lancet 397 (10291), 2284–2303. 10.1016/s0140-6736(21)00218-x 33848468

[B5] Cascella R. Chen S. W. Bigi A. Camino J. D. Xu C. K. Dobson C. M. (2021). The release of toxic oligomers from α-synuclein fibrils induces dysfunction in neuronal cells. Nat. Commun. 12 (1), 1814. 10.1038/s41467-021-21937-3 33753734 PMC7985515

[B6] Chen N. Han Y. P. Luo Y. Zhou Y. F. Hu X. J. Yu Y. (2018). Nanodiamond-based non-canonical autophagy inhibitor synergistically induces cell death in oxygen-deprived tumors. Mater. Horizons 5 (6), 1204–1210. 10.1039/c8mh00993g

[B7] Cui X. Zhang Z. Yang Y. Li S. Lee C. S. (2022). Organic radical materials in biomedical applications: state of the art and perspectives. Explor. (Beijing, China) 2 (2), 20210264. 10.1002/exp.20210264 PMC10190988 37323877

[B8] Du D. Su Y. L. Shang Q. Chen C. Tang W. K. Zhang L. (2022). Biomimetic synthesis of L-DOPA inspired by tyrosine hydroxylase. J. Inorg. Biochem. 234, 111878. 10.1016/j.jinorgbio.2022.111878 35660723

[B9] Fan Z. X. Jin H. Tan X. F. Li Y. Shi D. Wang Q. L. (2023). ROS-responsive hierarchical targeting vehicle-free nanodrugs for three-pronged Parkinson's disease therapy. Chem. Eng. J. 466, 143245. 10.1016/j.cej.2023.143245

[B10] Fujiwara M. Sun S. M. Dohms A. Nishimura Y. Suto K. Takezawa Y. (2020). Real-time nanodiamond thermometry probing *in vivo* thermogenic responses. Sci. Adv. 6 (37), eaba9636. 10.1126/sciadv.aba9636 32917703 PMC7486095

[B11] Gao Y. F. Zhai L. M. Chen J. P. Lin D. M. Zhang L. K. Yang H. (2024). Focused ultrasound-mediated cerium-based nanoreactor against Parkinson's disease via ROS regulation and microglia polarization. J. Control. Release 368, 580–594. 10.1016/j.jconrel.2024.03.010 38467194

[B12] Ghosh A. Langley M. R. Harischandra D. S. Neal M. L. Jin H. J. Anantharam V. (2016). Mitoapocynin treatment protects against neuroinflammation and dopaminergic neurodegeneration in a preclinical animal model of Parkinson’s disease. J. Neuroimmune Pharmacol. 11 (2), 259–278. 10.1007/s11481-016-9650-4 26838361 PMC4995106

[B13] Gong B. Zhang X. Zahrani A. A. Gao W. Ma G. Zhang L. (2022). Neural tissue engineering: from bioactive scaffolds and *in situ* monitoring to regeneration. Explor. (Beijing, China) 2 (3), 20210035. 10.1002/exp.20210035 PMC10190951 37323703

[B14] Gu M. J. Toh T. B. Hooi L. Lim J. J. Zhang X. Y. Chow E. K. H. (2019). Nanodiamond-mediated delivery of a G9a inhibitor for hepatocellular carcinoma therapy. Acs Appl. Mater. and Interfaces 11 (49), 45427–45441. 10.1021/acsami.9b16323 31718136

[B15] Hansson O. (2021). Biomarkers for neurodegenerative diseases. Nat. Med. 27 (6), 954–963. 10.1038/s41591-021-01382-x 34083813

[B16] Igarashi R. Yoshinari Y. Yokota H. Sugi T. Sugihara F. Ikeda K. (2012). Real-time background-free selective imaging of fluorescent nanodiamonds *in vivo* . Nano Lett. 12 (11), 5726–5732. 10.1021/nl302979d 23066639

[B17] Iranzo A. Fairfoul G. Ayudhaya A. C. N. Serradell M. Gelpi E. Vilaseca I. (2021). Detection of α-synuclein in CSF by RT-QuIC in patients with isolated rapid-eye-movement sleep behaviour disorder: a longitudinal observational study. Lancet Neurol. 20 (3), 203–212. 10.1016/s1474-4422(20)30449-x 33609478

[B18] Jomova K. Raptova R. Alomar S. Y. Alwasel S. H. Nepovimova E. Kuca K. (2023). Reactive oxygen species, toxicity, oxidative stress, and antioxidants: chronic diseases and aging. Archives Toxicol. 97 (10), 2499–2574. 10.1007/s00204-023-03562-9 PMC10475008 37597078

[B19] Li T. F. Li K. Wang C. Liu X. Wen Y. Xu Y. H. (2017). Harnessing the cross-talk between tumor cells and tumor-associated macrophages with a nano-drug for modulation of glioblastoma immune microenvironment. J. Control. Release 268, 128–146. 10.1016/j.jconrel.2017.10.024 29051064

[B20] Li T. F. Xu Y. H. Li K. Wang C. Liu X. Yue Y. (2019). Doxorubicin-polyglycerol-nanodiamond composites stimulate glioblastoma cell immunogenicity through activation of autophagy. Acta Biomater. 86, 381–394. 10.1016/j.actbio.2019.01.020 30654213

[B21] Lin Y. W. Raj E. N. Liao W. S. Lin J. Liu K. K. Chen T. H. (2017). Co-delivery of paclitaxel and cetuximab by nanodiamond enhances mitotic catastrophe and tumor inhibition. Sci. Rep. 7, 9814. 10.1038/s41598-017-09983-8 28852020 PMC5575327

[B22] Liu B. Qi Z. Chao J. (2023). Framework nucleic acids directed assembly of Au nanostructures for biomedical applications. Interdiscip. Med. 1 (1), e20220009. 10.1002/inmd.20220009

[B23] Liu Y. M. Chen S. Quan X. Yu H. T. (2015). Efficient electrochemical reduction of carbon dioxide to acetate on nitrogen-doped nanodiamond. J. Am. Chem. Soc. 137 (36), 11631–11636. 10.1021/jacs.5b02975 26322741

[B24] Malpartida A. B. Williamson M. Narendra D. P. Wade-Martins R. Ryan B. J. (2021). Mitochondrial dysfunction and mitophagy in Parkinson's disease: from mechanism to therapy. Trends Biochem. Sci. 46 (4), 329–343. 10.1016/j.tibs.2020.11.007 33323315

[B25] McMillan K. J. Murray T. K. Bengoa-Vergniory N. Cordero-Llana O. Cooper J. Buckley A. (2017). Loss of MicroRNA-7 regulation leads to α-synuclein accumulation and dopaminergic neuronal loss *in vivo* . Mol. Ther. 25 (10), 2404–2414. 10.1016/j.ymthe.2017.08.017 28927576 PMC5628933

[B26] Meng Z. Zhang Y. Yang L. Yuan F. Wang J. Chen J. (2024). Application of advanced biosensors in nervous system diseases. Interdiscip. Med. 2, e20240024. 10.1002/inmd.20240024

[B27] Ogawa M. Murae M. Gemba R. Irie T. Shimojima M. Saijo M. (2022). L-DOPA, a treatment for Parkinson's disease, and its enantiomer D-DOPA inhibit severe fever with thrombocytopenia syndrome virus infection *in vitro* . J. Infect. Chemother. 28 (3), 373–376. 10.1016/j.jiac.2021.11.005 34802888

[B28] Prasad E. M. Hung S. Y. (2020). Behavioral tests in neurotoxin-induced animal models of Parkinson’s disease. Antioxidants 9 (10), 1007. 10.3390/antiox9101007 33081318 PMC7602991

[B29] Russo M. J. Orru C. D. Concha-Marambio L. Giaisi S. Groveman B. R. Farris C. M. (2021). High diagnostic performance of independent alpha-synuclein seed amplification assays for detection of early Parkinson's disease. Acta Neuropathol. Commun. 9 (1), 179. 10.1186/s40478-021-01282-8 34742348 PMC8572469

[B30] Salaam A. D. Hwang P. T. J. Poonawalla A. Green H. N. Jun H. W. Dean D. (2014). Nanodiamonds enhance therapeutic efficacy of doxorubicin in treating metastatic hormone-refractory prostate cancer. Nanotechnology 25 (42), 425103. 10.1088/0957-4484/25/42/425103 25277401

[B31] Tenchov B. Sugimoto Y. Koynova R. Brueggemeier R. W. Lee R. J. (2012). Highly efficient cationic ethylphosphatidylcholine siRNA carrier for GFP suppression in modified breast cancer cells. Anticancer Res. 32 (7), 2563–2566. 10.1093/annonc/mds166 22753714 PMC3838928

[B32] Vijiaratnam N. Simuni T. Bandmann O. Morris H. R. Foltynie T. (2021). Progress towards therapies for disease modification in Parkinson's disease. Lancet Neurol. 20 (7), 559–572. 10.1016/s1474-4422(21)00061-2 34146514

[B33] Xie B. Xie H. (2024). Application of stimuli-responsive hydrogel in brain disease treatment. Front. Bioeng. Biotechnol. 12, 1450267. 10.3389/fbioe.2024.1450267 39091971 PMC11291207

[B34] Yang T. C. Chang C. Y. Yarmishyn A. A. Mao Y. S. Yang Y. P. Wang M. L. (2020). Carboxylated nanodiamond-mediated CRISPR-Cas9 delivery of human retinoschisis mutation into human iPSCs and mouse retina. Acta Biomater. 101, 484–494. 10.1016/j.actbio.2019.10.037 31672582

[B35] Zhang J. Zhao M. Y. Yan R. Liu J. Maddila S. Junn E. S. (2021). MicroRNA-7 protects against neurodegeneration induced by α-synuclein preformed fibrils in the mouse brain. Neurotherapeutics 18 (4), 2529–2540. 10.1007/s13311-021-01130-6 34697773 PMC8804150

