# Supplementary material for: Nanodiamond-mediated delivery of microRNA-7 for the neuroprotection of dopaminergic neurons
Source: Front Bioeng Biotechnol. 2025 Jan 8;12:1480573. doi: 10.3389/fbioe.2024.1480573 (PMC11751053; doi:10.3389/fbioe.2024.1480573)
Supplement: Supplementary file 1 [file DataSheet1.docx]

Supplementary Material


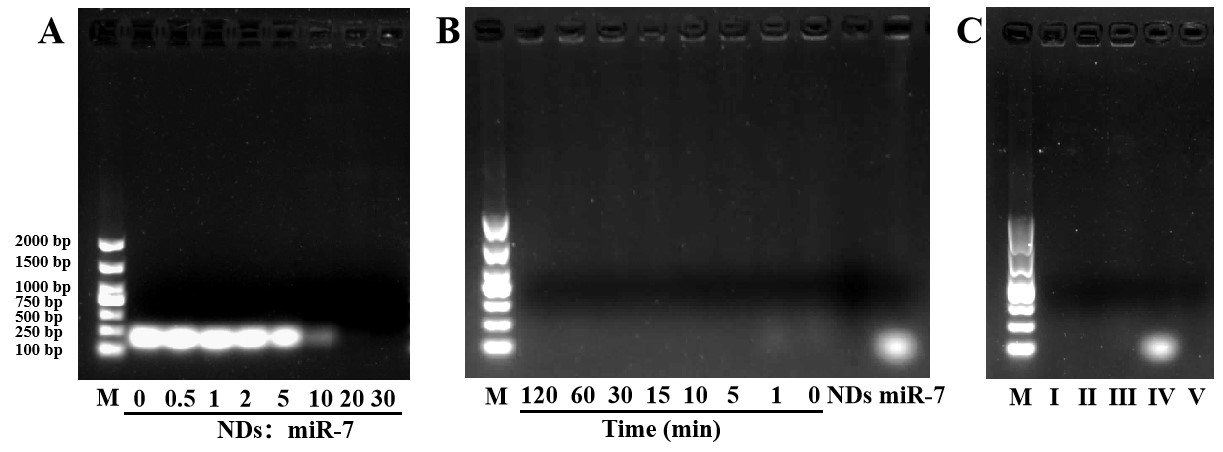


**Supplementary Figure 1. Synthesis of N-7.** (A) Gel retardation assays at mass ratios ranging from 0 to 30. M, marker. (B) Gel retardation assays at different time ranging from 0 to 120 minutes. (C) Gel retardation assays under different conditions. Ⅰ. Mix NDs and miR-7 for 10 minutes. Ⅱ. Supernatant of N-7. Ⅲ. Immediate loading of the mixture of NDs and miR-7 onto the agarose Gel. Ⅳ. Supernatant of miRNA. Ⅴ. Supernatant of NDs.


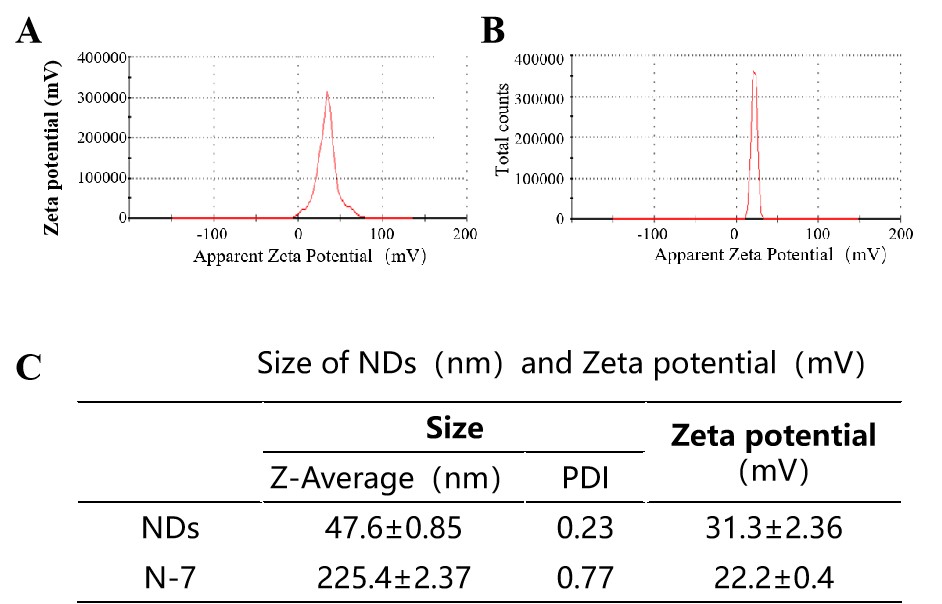


**Supplementary Figure 2. Characterization of NDs and N-7.** Zeta potential of NDs (A) and N-7 (B) were measured by dynamic light scattering. (C) Size and Zeta potential of NDs and N-7 were measured by dynamic light scattering.


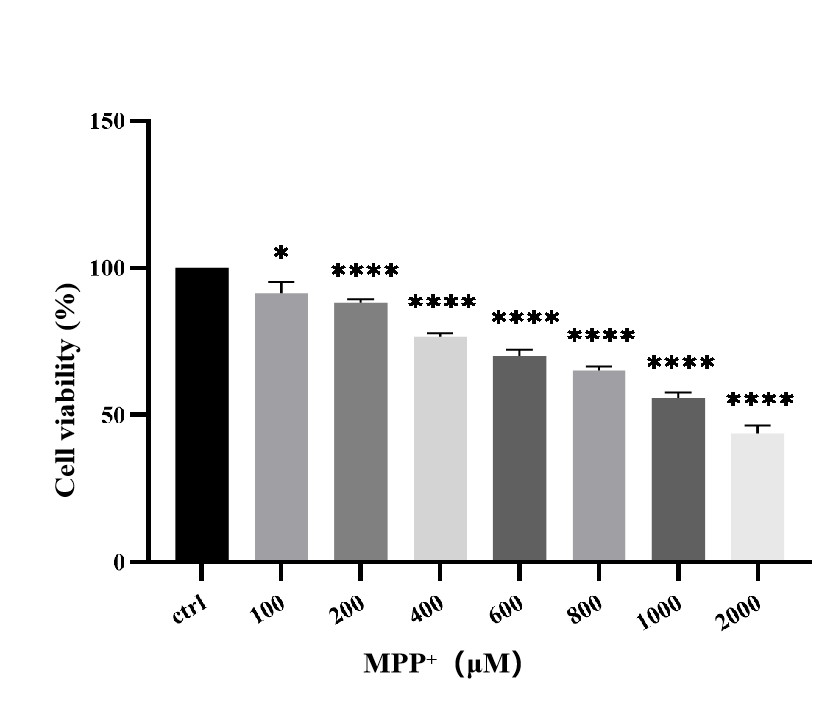


**Supplementary Figure 3. Cell viability of SH-SY5Y cells after being treated with different concentrations of MPP^+^.**

**Supplementary Table 1. Sequences of miRNA in human and mouse**

| **miRNA** | **sequences** | |
| --- | --- | --- |
| has-miR-7 mimic | sense  antisense | 5’-UGGAAGACUAGUGAUUUUGUUGUU-3’  5’-CAACAAAAUCACUAGUCUUCCAUU-3’ |
| has-miR-7 Inhibitor | sense  antisense | 5’-AACAACAAAAUCACUAGUCUUCCA-3’  / |
| mmu-miR-7 mimic | sense  antisense | 5’-UGGAAGACUAGUGAUUUUGUUGU-3’  5’-AACAAAAUCACUAGUCUUCCAUU-3’ |
| NC | sense  antisense | 5’-UUCUCCGAACGUGUCACGUTT-3’  5’-ACGUGACACGUUCGGAGAATT-3’ |

**Supplementary Table 2. Sequences of primer in human**

| **Gene** | **Forward Primers （5’-3’）** | **Reverse Primers（5’-3’）** |
| --- | --- | --- |
| *miR-7* | CGCGTGGAAGACTAGTGATTTT | AGTGCAGGGTCCGAGGTATT |
| *U6* | ACACGCACAAACGAGAAAGG | AGTGCAGGGTCCGAGGTATT |
| *TH* | AGTCAGTCTACTTCGTGTCTGAGA | GCGTGTACGGGTCGAACT |
| *SNCA* | AATTCTGGAAGATATGCCTGTGGAT | GGATGGAACATCTGTCAGCAGAT |
| *ACTB* | AGATTACTGCTCTGGCTCCTAGC | ACTCATCGTACTCCTGCTTGCT |
